# Risk of future cardiovascular diseases in different years postpartum after hypertensive disorders of pregnancy: A systematic review and meta-analysis

**DOI:** 10.1097/MD.0000000000029646

**Published:** 2022-07-29

**Authors:** Jarawee Sukmanee, Tippawan Liabsuetrakul

**Affiliations:** a Department of Epidemiology, Faculty of Medicine, Prince of Songkla University, Hat Yai, Songkhla, Thailand; b Department of Obstetrics and Gynecology, Faculty of Medicine, Prince of Songkla University, Songkhla, Thailand.

**Keywords:** cardiovascular diseases, hypertension, hypertension disorders of pregnancy, preeclampsia

## Abstract

**Objective:**

There are limited data on the optimal timing and frequency of postpartum follow-up visits after hypertensive disorders of pregnancy (HDP) for primary prevention and early detection of cardiovascular diseases (CVDs) in high-risk women. We aimed to evaluate the risk of cardiovascular outcomes later in life in women with prior HDP in different years postpartum and in preeclamptic women with severe features, or early onset of preeclampsia.

**Methods:**

We searched MEDLINE, Cochrane Library, Web of Science, and Scopus without language restriction for relevant articles published from inception to March 16, 2022. We included prospective and retrospective cohort studies assessing hypertension, ischemic heart disease, heart failure, venous thromboembolism, peripheral vascular disease, stroke, dementia, composite cardiovascular and/or cerebrovascular diseases, and mortality after 6 weeks postpartum, in women with prior HDP compared with controls. Two authors independently selected and appraised the studies. Article quality was independently assessed using the Newcastle-Ottawa Scale (NOS). Random-effect models were used for meta-analysis. Stratified analyses based on years postpartum, severity, and onset of preeclampsia were performed.

**Results:**

We included 59 studies for qualitative review, of which 56 were included in quantitative meta-analysis, involving 1,262,726 women with prior HDP and 14,711,054 controls. Women with prior HDP had increased risks of hypertension (relative risk [RR] 3.46, 95% confidence interval [CI]: 2.67–4.49), ischemic heart disease (RR 2.06, 95% CI: 1.38–3.08), and heart failure (RR 2.53, 95% CI: 1.28–5.00) later in life, compared with those with normotensive pregnancies. The risk of hypertension was highest during 5 years postpartum (RR 5.34, 95% CI: 2.74–10.39). Compared with normotensive pregnancies, the risk of future CVDs significantly increased in preeclamptic women.

**Discussion:**

A history of HDP is associated with approximately 2- to 4-fold increase in the risk of CVDs. Screening for CVDs and their risk factors in women with prior HDP since delivery, especially the first 5 years after delivery is suggested for early detection and appropriate management. Evidence on the risks of CVDs in preeclampsia with severe features and early onset of preeclampsia is limited due to having few studies and high heterogeneity.

**Funding:**

The Royal Golden Jubilee PhD Program–RGJ (PHD/0183/2561); Thailand Science Research and Innovation (TSRI) Research Career Development Grant–RSA (RSA6180009); Targeted Research Grants Program of the Faculty of Medicine, Prince of Songkla University, Thailand.

**Registration:**

CRD42020191550

## 1. Introduction

Cardiovascular diseases (CVDs) are a group of conditions affecting the heart and/or blood vessels, such as ischemic heart disease, venous thromboembolism, peripheral artery disease, and stroke.^[1]^ In 2019, CVDs accounted for 17.9 million deaths globally, of which 80% occurred in low- and middle-income countries.^[2]^ CVDs have also become a leading cause of death in Thailand for decades.^[3]^ Well-known risk factors for CVDs include older age, family history of CVDs, smoking, hypertension, diabetes mellitus, and dyslipidemia.^[4]^ However, there are several risk factors that are specific for women and one of them is hypertensive disorders of pregnancy (HDP).^[5]^

HDP are the most common medical complications in pregnancy and roughly affect 5% to 8% of all pregnant women.^[6]^ Among HDP, preeclampsia (hypertension plus proteinuria) is the largest contributor to maternal and neonatal morbidity.^[7,8]^ Adverse maternal and neonatal outcomes are more likely to occur in women with eclampsia, preeclampsia superimposed on chronic hypertension, preeclampsia with severe features (severe hypertension and/or significant end-organ injury),^[8,9]^ and early-onset preeclampsia (developed before 34 completed weeks of gestation).^[10]^ Women with prior preeclampsia are also associated with an approximately 2- to 4-fold increased risk for developing hypertension and CVDs later on in life in 4 previous systematic reviews.^[11–14]^ Although the association between future CVDs and HDP is well-established, previous studies have not focused on the severity of HDP or risks in different years after index pregnancy. This is important as there are the changes of cardiovascular risks in different years after HDP.^[15]^

According to immediate postpartum care, women with pregnancy complicated by HDP are usually advised to measure blood pressure at 3 to 10 days postpartum and to visit routine postpartum care at 6 weeks postpartum.^[16–18]^ National guidelines from the United States, United Kingdom, Norway, and Netherlands recommend the monitoring of blood pressure after HDP.^[5,16–20]^ These guidelines advise counseling women about the higher risk of CVDs later in life, as well as in regards to a healthy life style including exercise, smoking cessation, balanced diet, and optimal weight. Nevertheless, the timing of the postpartum follow-up visit varies from 3 to 5 months postpartum up to screening at the age of 50 and most guidelines do not differentiate between follow-up durations on the basis of the severity and onset of preeclampsia.^[5,16,18,19]^

To date, there is insufficient information on the progression after HDP to future CVDs. The optimal timing and frequency of postpartum follow-up visits in women with prior HDP are still unknown. A systematic review evaluating the risk of future CVDs in different years postpartum can provide information on the natural history and is useful for the development of CVDs screening guidelines in women with prior HDP. Moreover, identifying women at risk of future CVDs is beneficial for promoting primary prevention and early detection of CVDs in these women. This systematic review aimed to evaluate the risk of cardiovascular outcomes later in life in women with prior HDP, in regards to different postpartum years, preeclampsia with severe features, and early onset of preeclampsia.

## 2. Methods

### 2.1. Search strategy

This systematic review was conducted in accordance with the Preferred Reporting Items for Systematic Reviews and Meta-analysis (PRISMA) and Meta-Analyses and Systematic Reviews of Observational Studies (MOOSE).^[21,22]^ The review protocol was registered in the PROSPERO International Prospective Register of Systematic Reviews (CRD42020191550).

We searched articles published in any language in MEDLINE, Cochrane Library, Web of Science, and Scopus from inception to March 16, 2022. Detailed search strategies are provided in Supplemental Digital Content, http://links.lww.com/MD/G949. For those articles which full texts were not available, authors were contacted to ask for the full texts.^[23,24]^ Articles in languages other than English were translated using Google Translate. Duplicated articles were identified and removed using Zotero software before assessing the remaining articles.

### 2.2. Study selection

We included retrospective and prospective cohort studies assessing cardiovascular outcomes after 6 weeks postpartum in women with prior HDP compared with controls. HDP was classified and defined in regard to American College of Obstetricians and Gynecologists (ACOG) guidelines.^[25]^ Women with prior HDP, as the exposure, included gestational hypertension and preeclampsia, not chronic hypertension. Gestational hypertension is defined as “*a systolic blood pressure of 140 mm Hg or more or a diastolic blood pressure of 90 mm Hg or more, or both detected after 20 weeks of gestation in a woman with a previously normal blood pressure*.” Preeclampsia is defined as gestational hypertension with proteinuria that severe features include systolic blood pressure of 160 mm Hg or more, or diastolic blood pressure of 110 mm Hg or more, thrombocytopenia, impaired liver function, renal insufficiency, pulmonary edema, new-onset headache unresponsive to medication, and visual disturbances. Controls were defined as women with prior normotensive pregnancies, preeclampsia without severe features, or late-onset preeclampsia (developed after 34 completed weeks of gestation) depending on individual included studies. Case-control studies were excluded due to selection and recall biases. There was no restriction based on the severity of preeclampsia or duration of follow-up.

Cardiovascular outcomes in this review included hypertension (blood pressure ≥ 140/90 mm Hg), ischemic heart disease, heart failure, venous thromboembolism, peripheral vascular disease, stroke, dementia, cardiovascular and/or cerebrovascular diseases, cardiovascular mortality, and all-cause mortality. Ischemic heart disease included coronary heart disease, myocardial infarction, angina pectoris, coronary artery bypass grafting, coronary angioplasty, and balloon dilatation or stent placement. Venous thromboembolism included deep venous thrombosis and pulmonary embolism. Stroke included both hemorrhagic and ischemic cerebrovascular accidents. Composite cardiovascular and/or cerebrovascular diseases included any cardiovascular and cerebrovascular diseases. If there were multiple publications based on the same cohort, data from the study with the most comprehensive information and largest sample size, in descending priority order, were selected and extracted to avoid overlapping in data.

Two authors (JS and TL) independently screened the title and abstract of all search results in Rayyan software. Full texts of potentially relevant articles were further retrieved and assessed for eligibility by the same authors. Disagreements were resolved through discussion between 2 authors. The number of included and excluded articles was reported using the PRISMA flow diagram.

### 2.3. Data extraction and methodological assessment

Data were extracted by 2 authors independently. Extracted data included year of publication, country, study design and setting, inclusion and exclusion criteria, ascertainment of exposures and outcomes, outcomes per group, sample size of each group, follow-up duration, baseline characteristics of participants (age, parity, family history of HDP and CVDs, underlying diseases, smoking status, and body mass index), and covariates used for adjustment or matching.

Two authors assessed the quality of included articles independently using the Newcastle-Ottawa Scale (NOS).^[26]^ The NOS consisted of 8 items in 3 key domains with a maximum score of 9 stars: selection of study groups (four stars), comparability of groups (two stars), and ascertainment of outcomes (three stars). The detailed criteria for the NOS are provided in Supplemental Digital Content, http://links.lww.com/MD/G949. Studies with NOS scores of 0 to 3, 4 to 6, and 7 to 9 were classified as having low, moderate and high quality, respectively. Grading of Recommendations Assessment, Development and Evaluation (GRADE) methods were carried out based on 5 domains, namely risk of bias, imprecision, inconsistency, indirectness, and publication bias.^[27]^ The quality of evidence for each outcome was then presented as very low, low, moderate or high certainty in the summary of findings table.

### 2.4. Statistical analysis

We used an inverse variance method to calculate the effect size in terms of pooled relative risk (RR) at a 95% confidence interval (CI). A random-effect model was chosen as a substantial variation in study population and methodology was expected. A continuity correction of 0.1 was added to the number of events in studies with zero events. Meta-analyses of each cardiovascular outcome were stratified to assess the associations among: (1) women with prior HDP pregnancy, (2) women with preeclampsia with severe features only, (3) women with preeclampsia without severe features only, and (4) women with early-onset preeclampsia only, in comparison with women with prior normotensive pregnancies. Additional stratified analyses based on different years postpartum at ≤ 5 years, 6 to 10 years, 11–15 years, and > 15 years after index pregnancy were performed. Due to no standard years postpartum recommended for following up women with prior HDP for future risk of cardiovascular diseases, we used the 25th percentiles of known average years from the included studies, accounted for 5 years for stratification.

Heterogeneity was assessed using I^2^ test, where I^2^ values more than 50% was considered as having substantial heterogeneity.^[28]^ If there was a substantial heterogeneity, a sensitivity analysis was performed considering the outlier and influence studies.^[29]^ Possible publication bias was estimated by funnel plot visualization and the arcsine-Thompson test when there were at least ten studies included.^[30]^ A *P* value of <.05 was considered as statistical significance for all pooled estimates. Statistical analyses were performed with R version 4.0.4 (2020 The R Foundation for Statistical Computing, Vienna, Austria)^[31]^ using the “meta”^[32]^ and “dmetar”^[33]^ packages.

## 3. Results

### 3.1. Study identification and study characteristics

**Figure 1.**
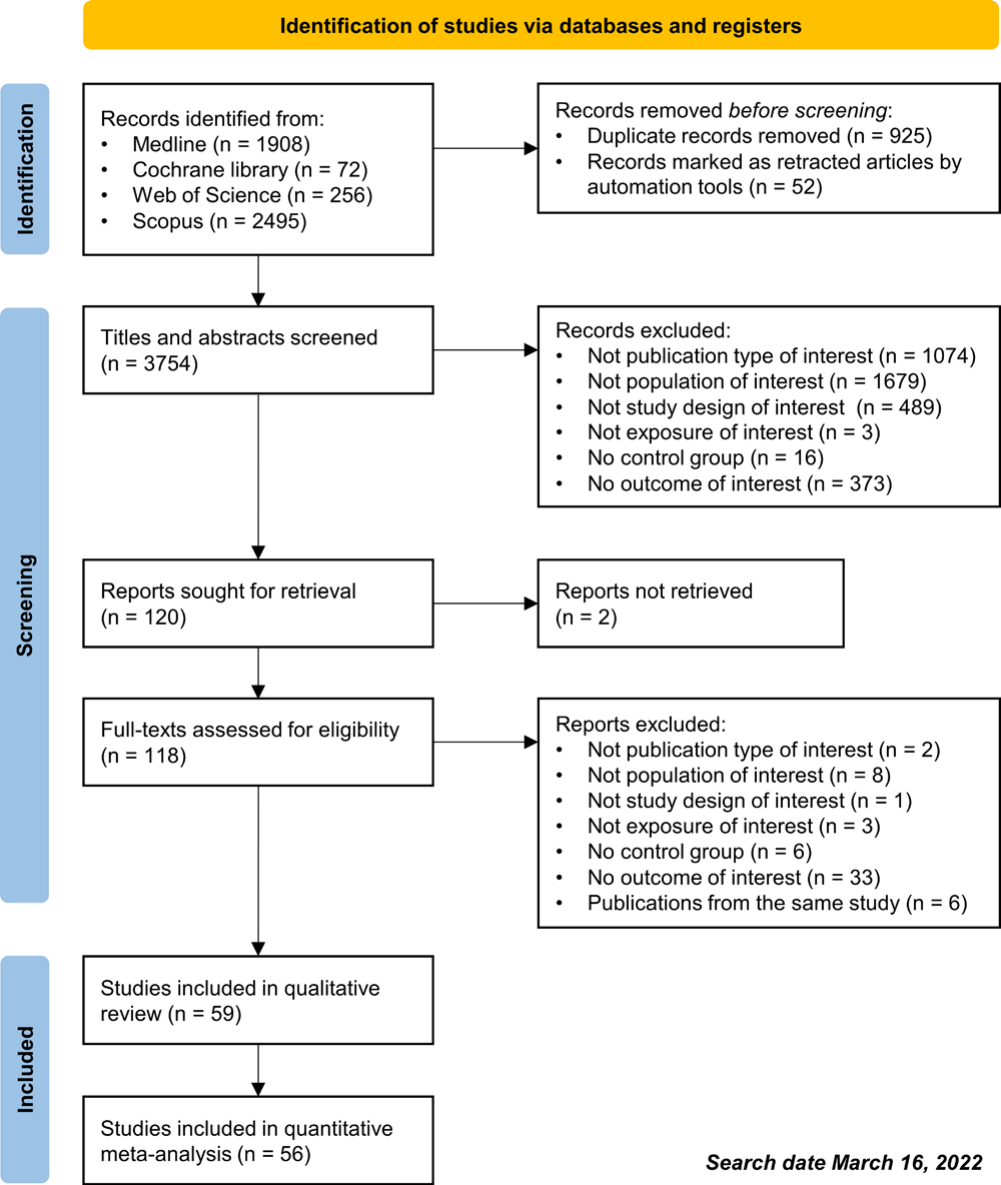
PRISMA flow diagram.

Study identification and selection processes in the PRISMA diagram are shown in Figure 1. The initial searches result in 3754 titles and abstracts. After screening based on eligibility criteria, 59 included studies were qualitatively reviewed, and 56 of them were quantitatively reviewed in the meta-analysis. Table 1 presents the characteristics of the studies included. There were 59 studies involving 1,262,726 women with prior HDP and 14,711,054 controls. These studies were conducted in Europe (27 studies),^[35,39,40,42,45,46,48,50,52,54–57,60,61,64,66,68,70,73,76,79,82–84,88,92]^ North America (14 studies),^[34,36–38,51,53,58,59,65,69,72,77,81,87]^ Asia (12 studies),^[41,47,49,62,63,67,71,74,78,80,89,90]^ and other regions of the world.^[43,44,75,85,86,91]^ The study design of the studies included was the retrospective cohort for 36 of them^[34,35,37,38,41,44,46,49,52–56,63–68,70–72,74,76–82,85,87–90,92]^ and the prospective cohort for the remaining 23 studies.^[36,39,40,42,43,45,47,48,50,51,57–62,69,73,75,83,84,86,91]^ In 23 studies, only women with preeclampsia were included^[36,38–40,44–48,50,59–61,66,67,71,74,76,82,84–87]^ and the other 36 studies included women with gestational hypertension and preeclampsia.^[34,35,37,41–43,49,51–58,62–65,68–70,72,73,75,77–81,83,88–92]^ The control subjects in all studies were women with prior normotensive pregnancies, except 1 study that compared women who had early-onset preeclampsia to those with late-onset preeclampsia^[74]^ which was not quantitatively analyzed in this meta-analysis. Mean or median follow-up duration was reported in 39 studies, and its weighted average was 11.6 years postpartum.

**Table 1:** Characteristics of included studies.

Author, year	Country	Study design	Exposure	No. of exposed	No. of control	Average duration (y)	Outcome
Kestenbaum, 2003^[34]^	United States	Retrospective cohort	Gestational hypertension, mild preeclampsia, severe preeclampsia	31,239	92,902	7.8 (mean)	Myocardial infarction, venous thromboembolism
Lykke, 2009*^[35]^	Denmark	Retrospective cohort	Gestational hypertension, mild preeclampsia, severe preeclampsia	41,275	741,012	14.6 (median)	**Hypertension, myocardial infarction**, heart failure, **venous thromboembolism, stroke**
Edlow, 2009^[36]^	United States	Prospective cohort	Preeclampsia	74	127	0.6 (mean)	Hypertension
Garovic, 2010^[37]^	United States	Retrospective cohort	Hypertensive pregnancy, preeclampsia	643	3421	NS	Hypertension, myocardial infarction, stroke
Mongraw-Chaffin, 2010^[38]^	United States	Retrospective cohort	Preeclampsia	481	13,922	37 (median)	Cardiovascular disease death
Melchiorre, 2011^[39]^	United Kingdom	Prospective cohort	Preeclampsia	64	78	NS	Hypertension
Drost, 2012^[40]^	Netherlands	Prospective cohort	Early-onset preeclampsia	339	332	9.9 (mean)	Hypertension
Zhao, 2012^[41]^	China	Retrospective cohort	Hypertensive pregnancy	581	3278	15.3 (mean)	Myocardial infarction, ischemic stroke, hemorrhagic stroke
Collen, 2013^[42]^	Sweden	Prospective cohort	Hypertensive pregnancy	50	55	NS	Hypertension
Callaway, 2013^[43]^	Australia	Prospective cohort	Hypertensive pregnancy	191	1926	NS	Hypertension
Shalom, 2013^[44]^	Israel	Retrospective cohort	Preeclampsia	2072	20,742	NS	Hypertension
Östlund, 2013^[45]^	Sweden	Prospective cohort	Severe preeclampsia	15	16	11.2 (mean)	Hypertension
Kvehaugen, 2014†^[46]^	Norway	Retrospective cohort	Preeclampsia	934	2011	15.1 (mean)	**Hypertension**, cardiovascular disease
Zhou, 2014^[47]^	China	Prospective cohort	Preeclampsia	651	2684	3.1 (mean)	Hypertension
Ghossein-Doha, 2014^[48]^	Netherlands	Prospective cohort	Preeclampsia	20	8	NS	Hypertension
Yeh, 2014‡^[49]^	Taiwan	Retrospective cohort	Hypertensive pregnancy	1260	5040	5.8 (median)	Hypertension, **cardiovascular disease**
Breetveld, 2014^[50]^	Netherlands	Prospective cohort	Preeclampsia	115	50	6.2 (median)	Hypertension, cardiovascular disease
Ehrenthal, 2015^[51]^	United States	Prospective cohort	Hypertensive pregnancy	31	40	NS	Hypertension
Behrens, 2016*^[52]^	Denmark	Retrospective cohort	Gestational hypertension, mild preeclampsia, severe preeclampsia	51,992	1,023,771	17.9 (mean)	**Cardiomyopathy, heart failure**
Cain, 2016^[53]^	United States	Retrospective cohort	Gestational hypertension, preeclampsia	24,221	265,973	4.8 (median)	Cardiovascular disease
Nelander, 2016^[54]^	Sweden	Retrospective cohort	Gestational hypertension, preeclampsia	382	2646	NS	Dementia, cardiovascular disease, stroke
Pérez-Adan, 2016^[55]^	Spain	Retrospective cohort	Hypertensive pregnancy	134	145	13.1 (mean)	Hypertension, stroke
Grandi, 2017§^[56]^	United Kingdom	Retrospective cohort	Hypertensive pregnancy	5399	141,349	4.7 (median)	Cardiovascular disease, **hypertension**
Timpka, 2017^[57]^	Netherlands	Prospective cohort	Hypertensive pregnancy	5520	49,068	NS	Hypertension
Dunietz, 2017^[58]^	United States	Prospective cohort	Hypertensive pregnancy	78	190	11.0 (median)	Hypertension
Best, 2017^[59]^	United States	Prospective cohort	Preeclampsia	130	288	13.1 (mean)	Hypertension
Ghossein-Doha, 2017^[60]^	Netherlands	Prospective cohort	Preeclampsia	107	41	5.6 (median)	Hypertension
Bokslag, 2018^[61]^	Netherlands	Prospective cohort	Early-onset preeclampsia	131	56	13.4 (mean)	Hypertension
Li, 2018^[62]^	Singapore	Prospective cohort	Hypertensive pregnancy	23	253	NS	Hypertension
Chen, 2018^[63]^	Taiwan	Retrospective cohort	Hypertensive pregnancy	29,186	116,744	5.7 (mean)	Heart failure
Bergen, 2018^[64]^	Netherlands	Retrospective cohort	Gestational hypertension, preeclampsia	300	4612	6 (median)	Hypertension
Theilen, 2018^[65]^	United States	Retrospective cohort	Hypertensive pregnancy	57,384	114,768	NS	All-cause mortality
Basit, 2018*^[66]^	Denmark	Retrospective cohort	Preeclampsia	58,410	1,119,595	21.1 (median)	**Dementia**
Kuo, 2018‡^[67]^	Taiwan	Retrospective cohort	Preeclampsia	1295	5180	9.8 (median)	Hypertension, **myocardial infarction, peripheral vascular disease**, heart failure, stroke
Egeland, 2018^[68]^	Norway	Retrospective cohort	Gestational hypertension, preeclampsia	3381	56,646	7.1 (mean)	Hypertension
Haas, 2019^[69]^	United States	Prospective cohort	Hypertensive pregnancy, preeclampsia	581	3355	3.0 (mean)	Hypertension
Haug, 2019†^[70]^	Norway	Retrospective cohort	Hypertensive pregnancy, gestational hypertension, preeclampsia	2119	21,766	18.0 (median)	**Cardiovascular disease, myocardial infarction, heart failure, stroke**
Amiri, 2019^[71]^	Iran	Retrospective cohort	Preeclampsia	355	2667	3.3 (median)	Hypertension
Smith, 2019^[72]^	Canada	Retrospective cohort	Hypertensive pregnancy, preeclampsia	375	130	NS	Hypertension
Honigberg, 2019^[73]^	United Kingdom	Prospective cohort	Hypertensive pregnancy	2808	217,216	7 (median)	Hypertension, myocardial infarction, heart failure, venous thromboembolism, peripheral artery disease, ischemic stroke
Ernawati, 2019^[74]^	Indonesia	Retrospective cohort	Early-onset preeclampsia	17	25∥	5.4 (mean)	Hypertension
Osoti, 2019^[75]^	Kenya	Prospective cohort	Hypertensive pregnancy	63	131	NS	Hypertension
Leon, 2019§^[76]^	United Kingdom	Retrospective cohort	Preeclampsia	25,554	1,277,811	9.2 (median)	**Cardiovascular disease**
Arnaout, 2019^[77]^	United States	Retrospective cohort	Gestational hypertension, preeclampsia	99,492	1,539,445	2.7 (median)	Myocardial infarction, heart failure, stroke
Huang, 2020‡^[78]^	Taiwan	Retrospective cohort	Hypertensive pregnancy	41,870	125,610	4.9¶	**Hypertension, heart disease, stroke**
Scheres, 2020^[79]^	Netherlands	Retrospective cohort	Hypertensive pregnancy, preeclampsia	264,135	1,624,849	13.7 (median)	Venous thromboembolism
Wagata, 2020^[80]^	Japan	Retrospective cohort	Hypertensive pregnancy	1585	31,827	NS	Hypertension
Garovic, 2020^[81]^	United States	Retrospective cohort	Hypertensive pregnancy	571	1142	35.9 (median)	Myocardial infarction, heart failure, stroke, dementia, hypertension
Bergman, 2020^[82]^	Sweden	Retrospective cohort	Preeclampsia	34,923	857,502	NS	Cardiovascular disease
Moe, 2020^[83]^	Norway	Prospective cohort	Gestational hypertension, early- and late-onset preeclampsia	116	94	1.1 (median)	Hypertension
Sharma, 2021^[84]^	Sweden	Prospective cohort	Preeclampsia	115	2319	49.9 (median)	Cardiovascular diseases
Mooij, 2021^[85]^	Tanzania	Retrospective cohort	Severe preeclampsia, eclampsia	24	72	NS	Hypertension
Ntlemo, 2021^[86]^	South Africa	Prospective cohort	Preeclampsia	150	163	NS	Hypertension
Nuckols, 2021^[87]^	United States	Retrospective cohort	Preeclampsia	23	38	1.5 (mean)	Hypertension
Oliver-Williams, 2022^[88]^	United Kingdom	Retrospective cohort	Gestational hypertension, preeclampsia	159,819	2,199,567	NS	Cardiovascular diseases, stroke, myocardial infarction, heart failure, cardiomyopathy, cardiac arrhythmia
Park, 2022^[89]^	Korea	Retrospective cohort	Gestational hypertension, preeclampsia or eclampsia	37,297	1,998,387	NS	Cardiac arrhythmia
Hung, 2022‡^[90]^	Taiwan	Retrospective cohort	Hypertensive pregnancy	13,617	54,468	NS	**Stroke**
Kennedy, 2022^[91]^	Australia	Prospective cohort	Gestational hypertension, preeclampsia	15	34	NS	Hypertension
van Baar, 2022^[92]^	Netherlands	Retrospective cohort	Gestational hypertension, preeclampsia	258,994	963,467	11.8 (mean)	Cardiovascular disease death

### 3.2. Study quality and risk of bias assessment

Assessments of study quality and risk of bias are demonstrated in Tables S1 and S2, Supplemental Digital Content, http://links.lww.com/MD/G949. Using the NOS, 40 studies were considered as high quality^[34,35,37,38,41,43,45,48,49,51–53,55,56,59,61,63–70,73–79,81–84,88–92]^ and the rest were identified as moderate quality.^[36,39,40,42,44,46,47,50,54,57,58,60,62,71,72,80,85–87]^ In 55 out of the 59 studies, women with HDP, served as the representative exposed participants, were selected from national databases or hospital medical record systems. Controls in all but 1 study were drawn from the same source as exposed women.^[72]^ Self-report or interview was used in 5 studies for measuring exposure^[57,62,71,73,80]^ and in 4 studies for measuring outcome.^[36,37,46,57]^ Definition of study outcome (hypertension) was not reported in 4 studies.^[39,42,45,50]^ Nine studies did not clearly state outcomes of interest in the methods of the studies.^[36,39,40,42,54,55,58,72,80]^ Only 21 studies clearly showed the adjustment for both key confounding factors (age, smoking, and body mass index) and other factors,^[37,38,43,45,51,53,54,56,57,59,61,64,68,69,73,77,81,84,88,89,91]^ while 8 studies did not adjust for any factors.^[44,50,60,71,85–87,92]^ Adequate follow-up duration was reported in 27 studies. Data regarding completeness of follow-up were unavailable in 25 studies^[37,38,43,44,46,49–51,53,54,56,57,59,60,63,65–68,72,76,78,88,90,91]^ and 7 of them had follow-up rates that were <80%.^[36,40,47,48,58,69,74]^ The summary of findings table of each outcome is presented in Table S3, Supplemental Digital Content, http://links.lww.com/MD/G949.

### 3.3. Risk of hypertension later in life

#### 3.3.1. Women with prior HDP versus normotensive pregnancies.

**Figure 2.**
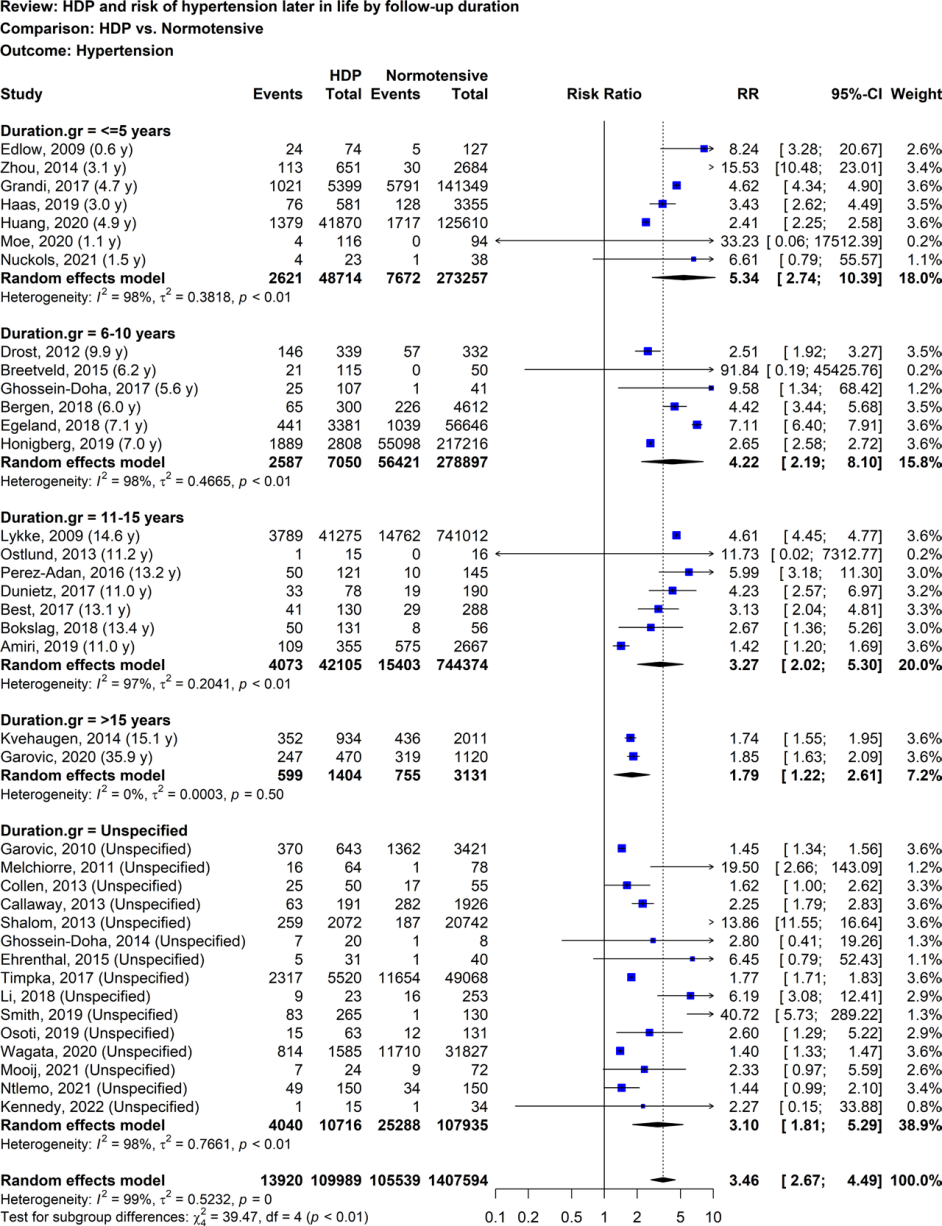
Forest plot of HDP and risk of hypertension later in life by follow-up duration. HDP = hypertensive disorder of pregnancy.

Of 56 studies included in the meta-analysis, 37 evaluated the risk of hypertension, later in life, in women with prior HDP in comparison to women with normotensive pregnancies involving 16 studies including only women with preeclampsia and 21 studies including women with gestational hypertension and preeclampsia (Fig. 2).^[35–37,39,40,42–48,50,51,55–62,64,68,69,71–73,75,78,80,81,83,85–87,91]^ Substantial heterogeneity was found in total meta-analysis (I^2^ = 99%). When stratified analyses based on different years postpartum were considered, substantial heterogeneities existed, except in the group with 15 years or more postpartum years. The findings of random-effect models showed that women with prior HDP had significantly higher risk of hypertension (RR 3.46, 95% CI: 2.67–4.49; I^2^ = 99%; 37 studies; 1,517,583 women; low certainty of evidence), compared to women without prior HDP, of which the RRs at different years postpartum at ≤ 5 years, 6 to 10 years, 11–15 years, and > 15 years were accounted for 5.34 (95% CI: 2.74–10.39; I^2^ = 98%; 7 studies; 321,971 women; very low certainty of evidence), 4.22 (95% CI: 2.19–8.10; I^2^ = 98%; 6 studies; 285,947 women; very low certainty of evidence), 3.27 (95% CI: 2.02–5.30; I^2^ = 97%; 7 studies; 786,479 women; low certainty of evidence), and 1.79 (95% CI: 1.22–2.61; I^2^ = 0%; 2 studies; 4535 women; low certainty of evidence), respectively. Similar findings were observed when a sensitivity analysis was performed.

#### 3.3.2. Women with prior preeclampsia in different severity and onset versus normotensive pregnancies.

**Figure 3.**
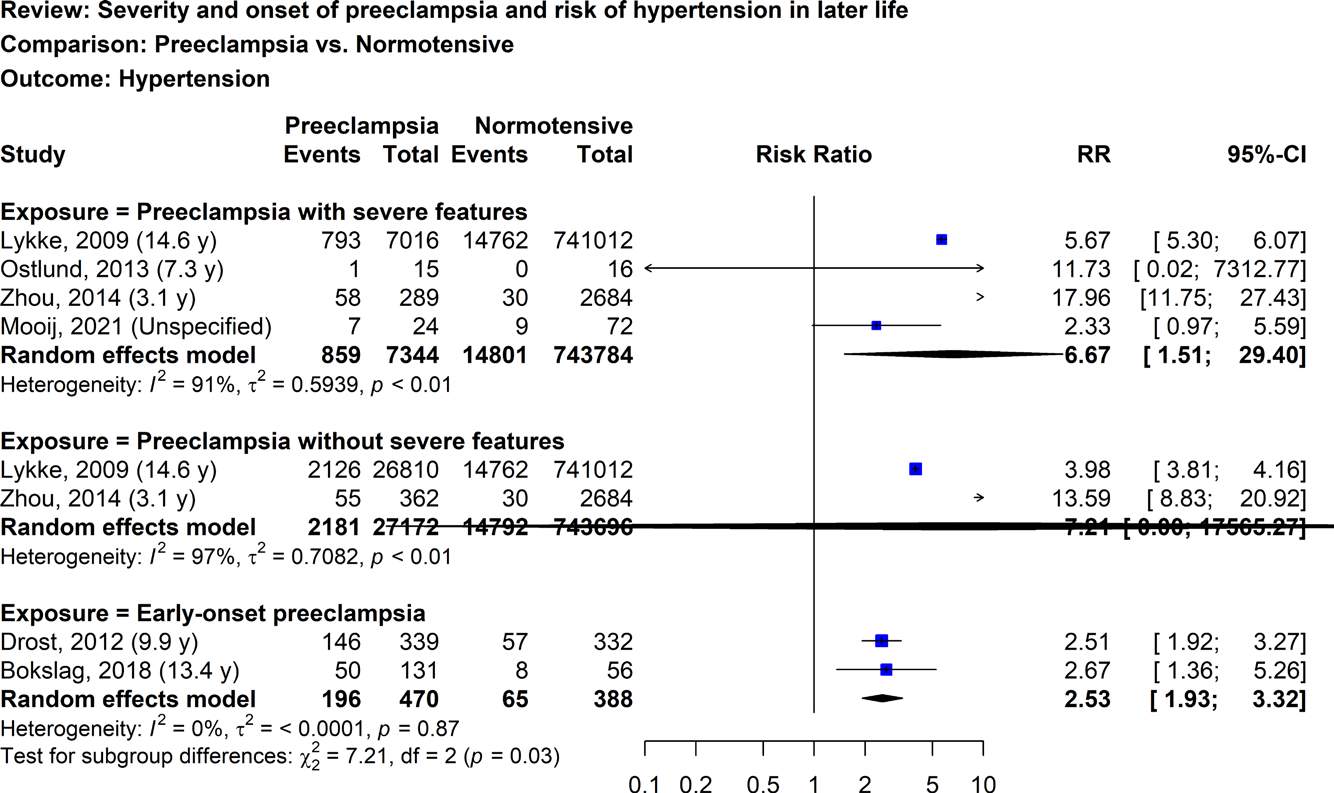
Forest plot of preeclampsia and risk of hypertension later in life by severity and onset of disease.

The risk of hypertension later in life in women with prior preeclampsia based on severity and onset in comparison to women with prior normotensive pregnancies is presented in Figure 3. Four studies evaluated the risk of hypertension later on in the life of women with prior preeclampsia that has severe features^[35,45,47,88]^ and 2 studies evaluated the same but for those without severe features,^[35,47]^ respectively. Women with prior preeclampsia with severe features had a greater risk of hypertension (RR 6.67, 95% CI: 1.51–29.40, I^2^ = 91%; 4 studies; 751,128 women; very low certainty of evidence) compared with normotensive pregnancies. Wide CI of the risk of hypertension in women without severe features was found. In 2 studies, the risk of hypertension in later life of women with prior early-onset preeclampsia was 2.53 (95% CI: 1.93–3.32, I^2^ = 0%; 2 studies; 858 women; low certainty of evidence).^[40,61]^

## 4. Risk of Cardiovascular Disease Later in Life

### 4.1. Women with prior HDP versus normotensive pregnancies

**Figure 4.**
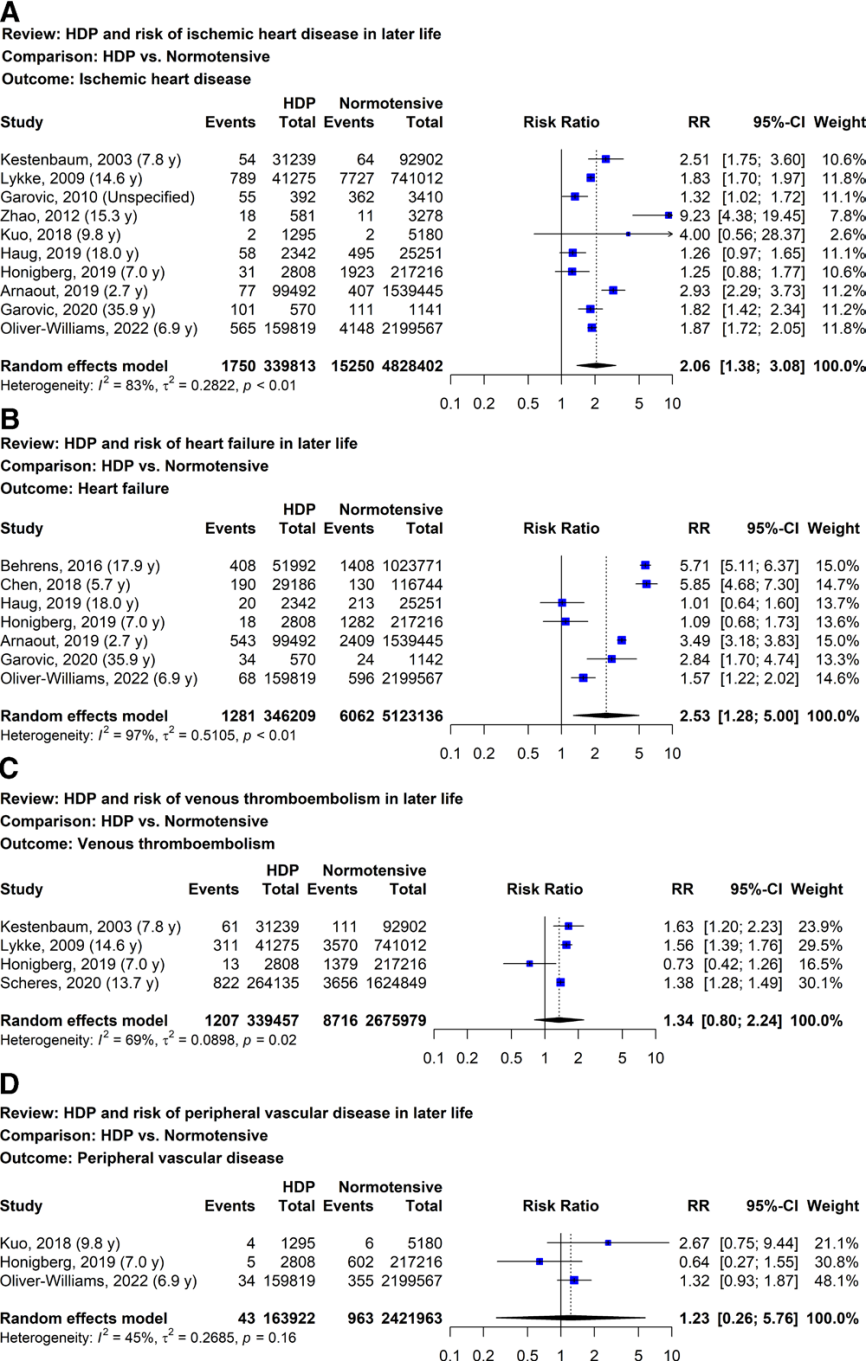
Forest plot of HDP and risk of ischemic heart disease (A), heart failure (B), venous thromboembolism (C), and peripheral vascular disease (D) later in life. HDP = hypertensive disorder of pregnancy.

The risk of 4 CVDs later in life including ischemic heart disease, heart failure, venous thromboembolism, and peripheral vascular disease is presented in Figure 4A–D. There were ten studies evaluating the risk of ischemic heart disease later in life for women with prior HDP compared with women with normotensive pregnancies (Fig. 4A).^[34,35,37,41,67,70,73,77,81,88]^ Women with prior HDP had approximately a twofold increase in risk (RR 2.06, 95% CI: 1.38–3.08, I^2^ = 83%; 10 studies; 5,168,215 women; low certainty of evidence). Substantial heterogeneity and similar results were also observed after we performed a sensitivity analysis. In 7 studies evaluating the risk of heart failure later in life, women with prior HDP were found to have significantly greater risk compared with women that have prior normotensive pregnancies (RR 2.53, 95% CI: 1.28–5.00, I^2^ = 97%; 7 studies; 5,469,345 women; very low certainty of evidence) as shown in Figure 4B.^[52,63,70,73,77,81,88]^ Sensitivity analysis did not change the heterogeneity and findings.

The risk of venous thromboembolism later in life in women with prior HDP was not significantly higher than in women with normotensive pregnancies in 4 studies (Fig. 4C) with RR of 1.34 (95% CI: 0.84–2.24, I^2^ = 69%; 4 studies; 3,015,436 women; very low certainty of evidence).^[34,35,73,79]^ In sensitivity analysis, the heterogeneity was not substantial and found that the risk of venous thromboembolism probably increased for women with prior HDP (RR 1.47, 95% CI: 1.20–1.80, I^2^ = 46%; 3 studies; 2,795,412 women; low certainty of evidence). Three studies evaluated the risk of peripheral vascular disease later in life in regards to women with prior HDP (Fig. 4D).^[67,73,88]^ The risk of peripheral vascular disease for women with prior HDP and normotensive pregnancies was not statistically different and a wide CI was also observed. The risks of ischemic heart disease, heart failure, and venous thromboembolism at different years postpartum showed high heterogeneity and a very low to low certainty of evidence.

### 4.2. Women with prior preeclampsia in different severity versus normotensive pregnancies

**Figure 5.**
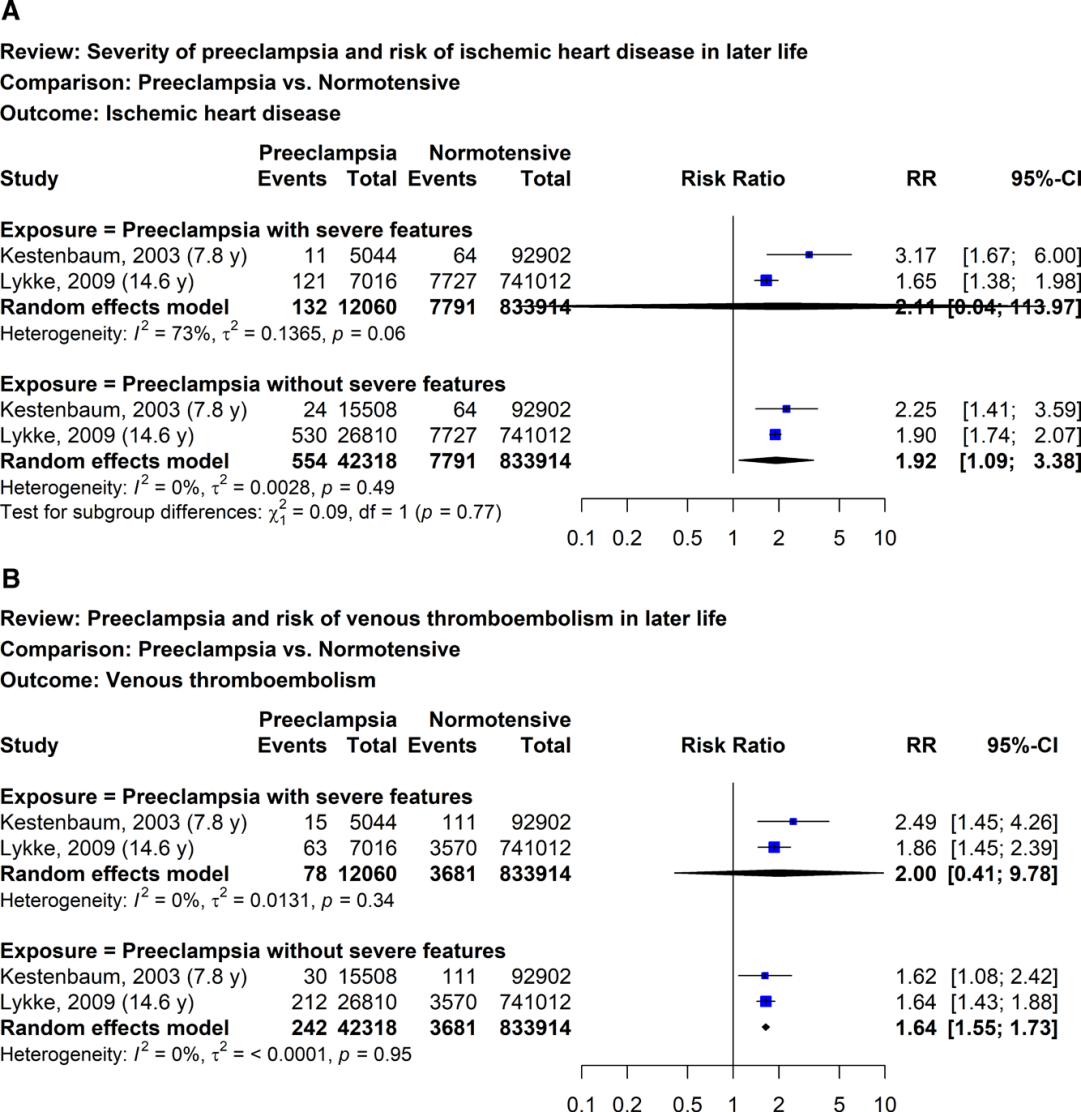
Forest plot of preeclampsia and risk of ischemic heart disease (A) and venous thromboembolism (B) later in life by disease severity.

Two studies evaluated the risks of ischemic heart disease (Fig. 5A) and venous thromboembolism (Fig. 5B) later in life for women with different severity of preeclampsia compared with women with prior normotensive pregnancies.^[34,35]^ Compared with women with prior normotensive pregnancies, the RRs of developing ischemic heart disease and venous thromboembolism in women with prior preeclampsia with severe features were 2.11 (95% CI: 0.04–113.97, I^2^ = 73%; 2 studies; 845,974 women; very low certainty of evidence) and 2.00 (95% CI: 0.41–9.78, I^2^ = 0%; 2 studies; 845,974 women; very low certainty of evidence), respectively. Women with prior preeclampsia without severe features had a slightly higher risk of developing ischemic heart disease (RR 1.92, 95% CI: 1.09–3.38, I^2^ = 0%; 2 studies; 876,232 women; low certainty of evidence) and venous thromboembolism (RR 1.64, 95% CI: 1.55–1.73, I^2^ = 0%; 2 studies; 876,232 women; low certainty of evidence) compared with women with prior normotensive pregnancies.

### 4.3. Risk of cerebrovascular disease later in life

**Figure 6.**
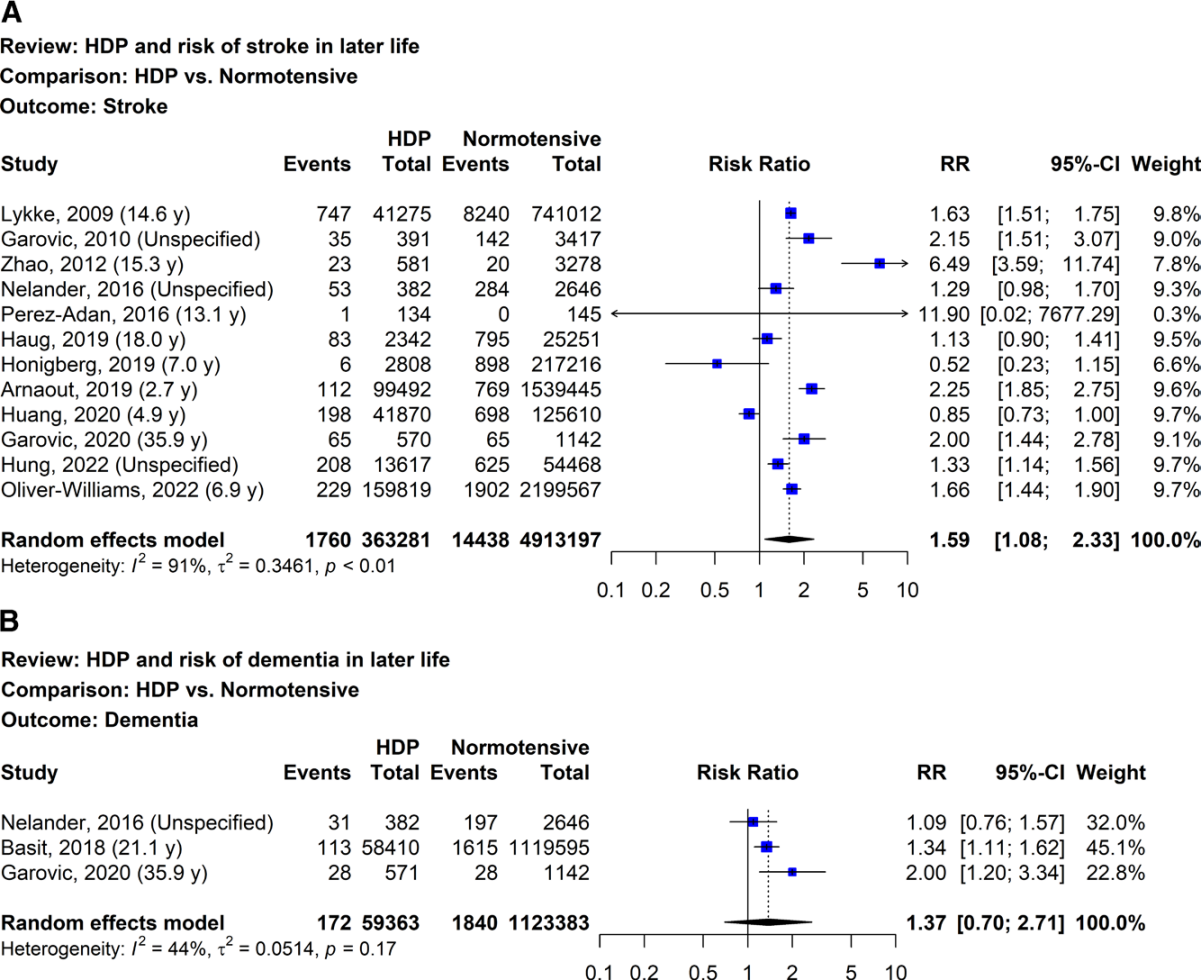
Forest plot of HDP and risk of stroke (A) and dementia (B) later in life. HDP = hypertensive disorder of pregnancy.

The risks of stroke and dementia later in life in women with prior HDP compared with women with normotensive pregnancies are demonstrated in Figure 6A and B, respectively. In regards to twelve studies evaluating the risk of stroke later in life, women with prior HDP had a greater risk compared with women with prior normotensive pregnancies (RR 1.59, 95% CI: 1.08–2.33, I^2^ = 91%; 12 studies; 5,276,478 women; low certainty of evidence).^[35,37,41,54,55,70,73,77,78,81,88,90]^ Furthermore, the risks of stroke at different years postpartum showed high heterogeneity and low certainty of evidence. Similar findings were detected when performing a sensitivity analysis. In 3 studies, the risk of dementia later in life in women with prior HDP in comparison to women with normotensive pregnancies was 1.37 (95% CI: 0.70–2.71, I^2^ = 44%; 3 studies; 1,182,746 women; very low certainty of evidence).^[54,66,81]^

### 4.4. Risk of composite cardiovascular and/or cerebrovascular diseases later in life

In 8 studies, the risks of composite cardiovascular and/or cerebrovascular diseases later on in life for women with prior HDP compared with women that had normotensive pregnancies are shown in Figure S1, Supplemental Digital Content, http://links.lww.com/MD/G949.^[41,49,53,70,76,82,84,88]^ We found that women with prior HDP had a higher risk of composite cardiovascular and/or cerebrovascular diseases (RR 1.91, 95% CI: 1.18–3.09, I^2^ = 97%; studies; 4,885,556 women; very low certainty of evidence). The risks of composite cardiovascular and/or cerebrovascular diseases at 6 to 10 years increased by 2.09 (95% CI: 1.19–3.66, 3 studies, I^2^ = 97.2%; studies; women; very low certainty of evidence). The estimated effects in each group were significantly different. When performing a sensitivity analysis, substantial heterogeneity and similar results were observed.

### 4.5. Risk of mortality later in life

Only 3 studies evaluated the risk of mortality later on in life for women with prior HDP compared with women that had normotensive pregnancies: 2 for cardiovascular-specific mortality and another for all-cause mortality. The pooled RR for cardiovascular-specific mortality in women with prior HDP was 2.81 (95% CI: 2.55–3.09, 2 studies, I^2^ = 0%; 2 studies; women; low certainty of evidence)^[38,92]^ and the RR for all-cause mortality reported in a study was 1.32 (95% CI: 1.27–1.36)^[65]^ comparing with normotensive pregnancies (Figure S2, Supplemental Digital Content, http://links.lww.com/MD/G949).

## 5. Discussion

Women with HDP had elevated risks of hypertension, ischemic heart disease, and heart failure later in life throughout postpartum years. Only hypertension had sufficient number of studies to stratify the analysis on its risk later in life for women with prior HDP in different periods of postpartum years. A history of HDP makes little or no difference regarding the risks of future venous thromboembolism, peripheral vascular disease, and dementia. Regardless of severity, women with prior preeclampsia had higher risks of hypertension, ischemic heart disease, and venous thromboembolism than what women with prior normotensive pregnancies had. These findings were based on studies of moderate and high quality. Nonetheless, substantial heterogeneity was found in most outcomes.

The findings of an increasing risk of hypertension later on in life for women with prior HDP in our review were consistent with previous systematic reviews only involving women with prior preeclampsia.^[11,14]^ This may suggest that HDP, regardless of gestational hypertension or preeclampsia, independently affects the risk of hypertension later in life.^[93]^ The highest effect of HDP on the risk of hypertension was during the first 5 years after pregnancy, then it decreased over time. A potential explanation is that hypertension prevalence increases with age^[94]^ that may be more dominant than a HDP history. Furthermore, the risk of hypertension after HDP still existed after performing a sensitivity analysis by excluding the predominated outcome effects studies. However, publication bias in the hypertensive outcome was indicated by an asymmetrical funnel plot (Figure S3, Supplemental Digital Content, http://links.lww.com/MD/G949) with a statistical significance in regards to the arcsine-Thompson test,^[30]^ suggesting that any interpretation should be done with caution. A strong association between a history of preeclampsia with severe features and the risk of future hypertension found in our review could be explained by the synergistic effect of the severity and onset of preeclampsia. However, the wide CI of effects may be due to the variations in women’s race and age at follow-up visit among included studies in our systematic review.^[35,45]^

The increasing risk of ischemic heart disease and heart failure later on in life for women with prior HDP in our systematic review was supported by the findings of previous systematic reviews.^[11–13]^ We also found that the risk of heart failure was higher than ischemic heart disease, which might be confounded by unmeasured CVD risk factors since only 1 included study adjusted for all identified factors including age, smoking, family history of CVDs, BMI, hypertension, diabetes mellitus, and dyslipidemia.^[56]^ The mechanistic association between HDP and future CVDs remains undetermined^[95,96]^; however, it may be hypothesized that HDP and CVDs generally share common risk factors. In addition, HDP may exaggerate prepregnancy risk factors or cause de novo endothelial injury and metabolic abnormalities possibly resulting in left ventricular hypertrophy,^[97]^ as an independent predictor of CVDs and mortality.^[98,99]^ The interpretation of risks of future ischemic heart disease and venous thromboembolism in women with or without severe features should be done with caution because of the small number of studies in each group and the wide CI in preeclampsia with severe features. We did not find a statistical difference in the risk of peripheral vascular disease for women with prior HDP and normotensive pregnancies. This was consistent with the result of a previous systematic review,^[12]^ but the estimated effect was imprecise since we could only include 2 studies for this outcome.

The RR of future stroke in women with prior HDP in our systematic review was similar to what was previously reported.^[11,13]^ Substantial heterogeneity between studies might be due to variation in research methodology. For example, 2 studies ascertained exposure status via interview,^[37,54]^ 1 study only explored the risk of ischemic cerebrovascular accident,^[73]^ and 1 study did not include all stroke-related International Classification of Diseases (ICD) codes regarding outcome.^[78]^ We also observed a positive association between history of HDP and the risk of dementia later in life, which was not assessed in the previous review. Nevertheless, the effects of HDP varied and it was possible that they made little or no difference to the risks of future stroke and dementia. Findings on the risk of composite cardiovascular and/or cerebrovascular diseases were consistent with the risks of specifically defined cardiovascular and cerebrovascular diseases in women with prior HDP.

To date, there have been limited data in regards to the risk of CVDs in women and the critical point of disease development after HDP, which may have different pathophysiology and effect of cardiovascular outcomes from preeclampsia. Our systematic review included more than 50 studies involving over 10 million women to assess various cardiovascular outcomes after HDP in different postpartum years. Moreover, potential risk of CVDs identified mostly during the first 5 years after HDP, preeclamptic women with severe features or early-onset of preeclampsia is an essential component for a strategy aiming to target high-risk women with appropriate policy of follow-up periods.

There are some limitations to our systematic review. First, the heterogeneity of effects was high in almost all outcomes. We found that sensitivity analysis based on the study quality score of the NOS was not suitable for the meta-analysis of observational studies due to questionable validity and reliability of the tool.^[100,101]^ Hence, we conducted a sensitivity analysis using outlier and influence diagnostics, which mostly contributed to similar results. This high heterogeneity may be due to different women’s characteristics across studies. There were a variation in race/ethnicity,^[43,44,75]^ study setting,^[41,57]^ as well as obstetric characteristics.^[53,56,69,77]^ Second, this systematic review used aggregate data for which not all potential confounding factors could be adjusted. Meta-analysis of individual participant data from studies with available data may successfully mitigate the heterogeneity and confounder issues. Third, the evaluation of publication bias was limited in scope as only the bias in hypertensive outcomes could be assessed. Fourth, the number of studies was too small to perform stratified analysis by severity and onset of preeclampsia in every cardiovascular outcome. Fifth, only 1 study comparing early- with late-onset preeclampsia on risk of future cardiovascular diseases which was not included in this meta-analysis and no study directly compared between preeclampsia with and without severe features. Finally, only available search databases, namely MEDLINE, Cochrane Library, Web of Science, and Scopus, were used which unpublished studies and some studies may not be retrieved.

## 6. Conclusion

Women with prior HDP had approximately a 2- to 4-fold increased risk for hypertension, ischemic heart disease, heart failure later in life, and cardiovascular mortality compared with those with normotensive pregnancies. The risk of future hypertension was highest during the first 5 years after delivery. However, the certainty of most evidence was very low and low due to limitation of study design, inconsistency, and imprecision. Influences of different severity and onset of preeclampsia are still inconclusive. Our findings emphasize the significance of CVD risks after HDP especially in women with preeclampsia with severe features, requiring proper screening after delivery for the early detection of subclinical CVDs, particularly during the 5 years after index HDP.

## Author contributions

Conceptualization: Jarawee Sukmanee, Tippawan Liabsuetrakul.

Data curation: Jarawee Sukmanee, Tippawan Liabsuetrakul.

Formal analysis: Jarawee Sukmanee.

Funding acquisition: Jarawee Sukmanee, Tippawan Liabsuetrakul.

Methodology: Jarawee Sukmanee, Tippawan Liabsuetrakul.

Supervision: Tippawan Liabsuetrakul.

Validation: Jarawee Sukmanee, Tippawan Liabsuetrakul.

Visualization: Jarawee Sukmanee.

Writing—original draft: Jarawee Sukmanee, Tippawan Liabsuetrakul.

Writing—review and editing: Jarawee Sukmanee, Tippawan Liabsuetrakul.

## Supplementary Material



## Footnotes

* Participants in these 3 studies were drawn from Denmark National Patient Register (NPR). Outcomes extracted from each study were given in boldface.

† Participants in these 2 studies were drawn from Norway Nord-Trøndelag Health (HUNT) Study. Outcomes extracted from each study were given in boldface.

‡ Participants in these 4 studies were drawn from Taiwan National Health Insurance Research Database (NHIRD). Outcomes extracted from each study were given in boldface.

§ Participants in these 2 studies were drawn from United Kingdom’s Clinical Practice Research Datalink (CPRD). Outcomes extracted from each study were given in boldface.

∥ Controls were women with late-onset preeclampsia.

¶ Calculated from the total number of women years.
