# Supplementary material for: Risk of future cardiovascular diseases in different years postpartum after hypertensive disorders of pregnancy: A systematic review and meta-analysis
Source: Medicine (Baltimore). 2022 Jul 29;101(30):e29646. doi: 10.1097/MD.0000000000029646 (PMC9333537; doi:10.1097/MD.0000000000029646)
Supplement: Supplementary file 1 [file medi-101-e29646-s001.pdf]

**Appendix 1** Searching strategies

**MEDLINE (PubMed)**

Search date: March 16, 2022

Results: 1,908

#1: "hypertension, pregnancy induced"[MeSH Terms] OR ("hypertension"[All Fields] AND "pregnancy induced"[All Fields]) OR "pregnancy-induced hypertension"[All Fields] OR ("gestational"[All Fields] AND "hypertension"[All Fields]) OR "gestational hypertension"[All Fields] OR ("hypertension, pregnancy induced"[MeSH Terms] OR ("hypertension"[All Fields] AND "pregnancy induced"[All Fields]) OR "pregnancy-induced hypertension"[All Fields] OR ("pregnancy"[All Fields] AND "induced"[All Fields] AND "hypertension"[All Fields]) OR "pregnancy induced hypertension"[All Fields]) OR ("pre eclampsia"[MeSH Terms] OR "pre eclampsia"[All Fields] OR "preeclampsia"[All Fields]) OR ("eclampsia"[MeSH Terms] OR "eclampsia"[All Fields] OR "eclampsias"[All Fields])

#2: "postpartum period"[MeSH Terms] OR ("postpartum"[All Fields] AND "period"[All Fields]) OR "postpartum period"[All Fields] OR "postpartum"[All Fields]

#3: "cardiovascular diseases"[MeSH Terms] OR ("cardiovascular"[All Fields] AND "diseases"[All Fields]) OR "cardiovascular diseases"[All Fields] OR ("cardiovascular"[All Fields] AND "disease"[All Fields]) OR "cardiovascular disease"[All Fields]

#4: #1 AND #2 AND #3

**Cochrane library**

Search date: March 16, 2022

Results: 72

#1: “gestational hypertension OR pregnancy induced hypertension OR preeclampsia OR eclampsia”

#2: “postpartum”

#3: “cardiovascular disease”

#4: (#1) AND (#2) AND (#3)

**Web of Science**

Search date: March 16, 2022

Results: 256

#1: ALL=(“gestational hypertension”) OR ALL=(“pregnancy induced hypertension”) OR ALL=(“preeclampsia”) OR ALL=(“eclampsia”)

#2: ALL=(“postpartum”)

#3: ALL=(“cardiovascular disease”)

#4: #1 AND #2 AND #3

#5: #1 AND #2 AND #3 AND DOCUMENT TYPES: (ARTICLE)

**Scopus**

Search date: March 16, 2022

Results: 2,495

#1: ( "gestational hypertension" OR "pregnancy induced hypertension" OR "preeclampsia" OR "eclampsia" ) AND "postpartum" AND "cardiovascular disease" AND ( LIMIT-TO ( SRCTYPE , "j" ) ) AND ( LIMIT-TO ( DOCTYPE , "ar" ) )

**Appendix 2** Criteria for the Newcastle-Ottawa Scale regarding star allocation to assess quality of studies

| **Criteria** | **Acceptable (star given)** | **Unacceptable (star not given)** |
|---|---|---|
| **Selection** | | |
| Representativeness of the exposed cohort | Population- or hospital-based | Selected group of women |
| Selection of the non-exposed cohort | Drawn from the same source as the exposed cohort | Drawn from a different source |
| Ascertainment of exposure | Secure record or structured interview | Written self-report |
| Demonstration that outcome of interest was not present at the start of the study | Demonstrated in exclusion criteria or analyzed in separated group | Did not demonstrate |
| **Comparability** | | |
| Comparability of cohorts on the basis of the design or analysis | Matched or adjusted for age, BMI, and smoking | Did not match or adjust for age, BMI, and smoking |
| | Matched or adjusted for any additional factor | Did not match or adjust for any additional factor |
| **Outcome** | | |
| Assessment of outcome | Directly measured or record linkage | Self-report or no information regarding definition of outcome |
| Follow-up long enough for outcomes to occur | Follow-up ≥ 5 years | Follow-up < 5 years or no information regarding follow-up duration |
| Adequacy of follow up of cohorts | Follow-up rate ≥ 80 % | Follow-up rate < 80 % or no information regarding follow-up rate |

BMI = body mass index

**Review: HDP and risk of any cardiovascular diseases in later life**

**Comparison: HDP vs. Normotensive**

**Outcome: Composite cardiovascular and cerebrovascular diseases**

| | HDP | | Normotensive | | | | | |
|---|---|---|---|---|---|---|---|---|
| **Study** | **Events** | **Total** | **Events** | **Total** | **Risk Ratio** | **RR** | **95%-CI** | **Weight** |
| Zhao, 2012 (15.3 y) | 40 | 581 | 31 | 3278 | | 7.28 | [4.59; 11.54] | 11.1% |
| Yeh, 2014 (5.8 y) | 68 | 1260 | 114 | 5040 | | 2.39 | [1.78; 3.20] | 12.2% |
| Cain, 2016 (4.8 y) | 265 | 24221 | 2269 | 265973 | | 1.28 | [1.13; 1.46] | 12.9% |
| Haug, 2019 (18.0 y) | 158 | 2342 | 1530 | 25251 | | 1.11 | [0.95; 1.30] | 12.8% |
| Leon, 2019 (9.2 y) | 861 | 25554 | 17763 | 1277811 | | 2.42 | [2.27; 2.59] | 13.0% |
| Bergman, 2020 (Unspecified) | 1053 | 34923 | 17362 | 857502 | | 1.49 | [1.40; 1.58] | 13.0% |
| Sharma, 2021 (42.1 y) | 35 | 115 | 499 | 2319 | | 1.41 | [1.06; 1.88] | 12.2% |
| Oliver-Williams, 2022 (6.9 y) | 1155 | 159819 | 9651 | 2199567 | | 1.65 | [1.55; 1.75] | 13.0% |
| **Random effects model** | **3635** | **248815** | **49219** | **4636741** | | **1.91** | **[1.18; 3.09]** | **100.0%** |

Heterogeneity: $I^2 = 97\%$, $\tau^2 = 0.3194$, $p < 0.01$

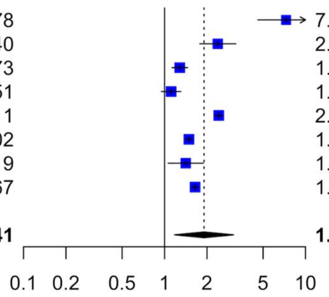

**Review: HDP and risk of mortality in later life**

**Comparison: HDP vs. Normotensive**

**Outcome: Mortality**

| Study | HDP Events | HDP Total | Normotensive Events | Normotensive Total | RR | 95%-CI | Weight |
|---|---|---|---|---|---|---|---|
| **Outcome = All-cause mortality** | | | | | | | |
| Theilen, 2018 (Unspecified) | 4722 | 57384 | 7172 | 114768 | 1.32 | [1.27; 1.36] | 35.9% |
| **Outcome = Cardiovascular mortality** | | | | | | | |
| Mongraw-Chaffin, 2010 (37.0 y) | 24 | 481 | 242 | 13922 | 2.87 | [1.91; 4.32] | 29.1% |
| van Baar, 2022 (11.8 y) | 369 | 258994 | 501 | 984237 | 2.80 | [2.45; 3.20] | 35.0% |
| **Random effects model** | **393** | **259475** | **743** | **998159** | **2.81** | **[2.55; 3.09]** | **64.1%** |
| Heterogeneity: $I^2 = 0\%$, $\tau^2 = < 0.0001$, $p = 0.91$ | | | | | | | |
| **Random effects model** | **5115** | **316859** | **7915** | **1112927** | **2.15** | **[0.70; 6.58]** | **100.0%** |
| Heterogeneity: $I^2 = 98\%$, $\tau^2 = 0.1847$, $p < 0.01$ | | | | | | | |
| Test for subgroup differences: $\chi^2_1 = 1502.42$, df = 1 ($p = 0$) | | | | | | | |

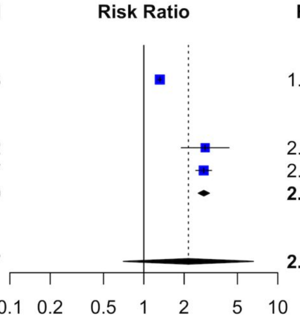

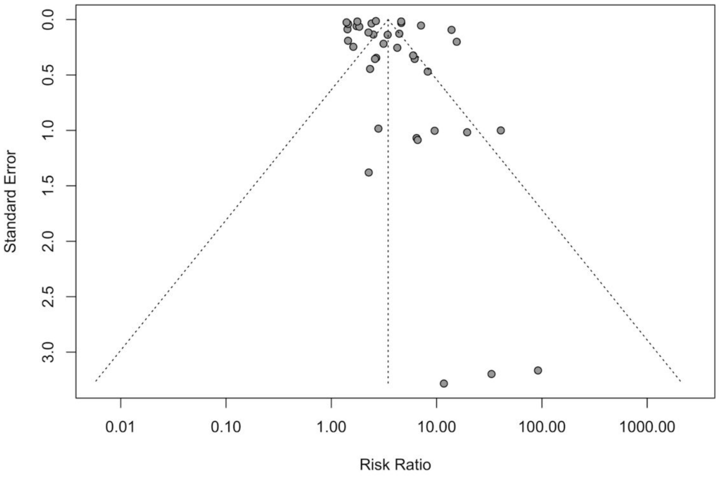
Standard Error
0.0
0.5
1.0
1.5
2.0
2.5
3.0
0.01
0.10
1.00
10.00
100.00
1000.00
Risk Ratio

**Supplementary Table 1** Quality assessment of included studies

| Author, year | Study population | Ascertainment of exposure | Ascertainment of outcome | Completeness of follow-up | Degree of adjustment* |
|---|---|---|---|---|---|
| Kestenbaum, 2003[61] | All singleton births recorded in Washington State Birth Events Record Database (BERD), 1987–1998 | ICD codes and diagnoses in birth certificate | ICD codes during hospitalization | No information | Age, parity, year of delivery, race/ethnicity, GDM, smoking, cesarean section, type of insurance used for the index pregnancy |
| Lykke, 2009[34] | All singleton deliveries in Denmark, January 1978 – October 2007 | ICD codes in National Patient Registry | ICD codes in National Patient Registry | 99.2% complete data | Age at delivery, year of delivery, preterm delivery, SGA, placental abruption, stillbirth, DM subsequent to the index pregnancy |
| Edlow, 2009[62] | Women enrolled in the Preeclampsia: Mechanisms and Consequences (PMC) study at the Hospital of the University of Pennsylvania, January 2006 – October 2006 | Maternal criteria for preeclampsia | Telephone interviews by the primary author | 70% of preeclampsia and 59% of controls completed the follow-up study | Race, BMI, parity, chronic hypertension |
| Garovic, 2010[63] | Women participating in the Family Blood Pressure Program (FBPP) study which consists of four different research networks in United States | Interviews by trained examiners | Self-reports and blood pressure measurement | No information | Race, network, family history of cardiovascular disease, education, DM, smoking, BMI, hypertension, dyslipidemia |

| | | | | | |
|---|---|---|---|---|---|
| Mongraw-Chaffin, 2010[64] | Women enrolled in the original Child Health and Development Studies (CHDS) cohort at the East Bay Area of California, 1959–1967 | Medical records abstraction | Results from the California Vital Status matches to CHDS files | No information | IUGR child, preexisting hypertension, age, BMI, current smoking at enrollment |
| Melchiorre, 2011[35] | Women visiting Fetal-Maternal Medicine Unit of St George's Hospital, United Kingdom, January 2008 – December 2010 | Diagnoses in medical records | No definition of outcome (hypertension) | 83.1% of preeclampsia and 100% of controls complete data | Age, ethnicity, gestational age at echocardiography |
| Drost, 2012[36] | Women registered in the obstetric database at the Isala Klinieken in Zwolle, Netherlands, 1991–2007 | Diagnoses in medical records | Blood pressure measurement | 83.9% of preeclampsia and 73.7% of controls complete data | Age, years postpartum, current smoking |
| Zhao, 2012[75] | Female employees giving birth at the Kailuan Medical Group, China, October 1976 – December 2008 | Delivery data filled by trained medical staff | Diagnoses in medical records | 83.7% of preeclampsia and 83.3% of controls complete data | Childbearing age, age at the 2006-2007 physical examination, BMI, blood pressure, fasting blood glucose, total cholesterol, triglycerides, LDL-C, HDL-C, hs-CRP |
| Collen, 2013[37] | Women giving birth at the Sahlgrenska University Hospital and Östra Hospital, Sweden, 1969–1973 | No information | No definition of outcome (hypertension) | 94.6% complete data | Age |
| Callaway, 2013[87] | Women delivering a single live birth at a major public hospital in South Brisbane, Australia, 1981–1983 | Diagnoses recorded by obstetricians | Blood pressure measurement | No information | Age, ethnicity, education, alcohol intake, number of cigarettes smoked, exercise, BMI |

| | | | | | |
|---|---|---|---|---|---|
| Shalom, 2013[88] | Women giving birth at the Soroka University Medical Center, Israel, January 1988 – December 1998 | Diagnoses in obstetric database | Diagnoses in Demog-ICD9 database | No information | None |
| Östlund, 2013[38] | Women giving birth at the Karolinska University Hospital, Sweden | Diagnoses in hospital records | No definition of outcome (hypertension) | 88.9% of preeclampsia and 94.1% of controls complete data | Age, parity, date of delivery |
| Kvehaugen, 2014[39] | Women participating in the phase 2 of the Nord-Trøndelag Health Study (HUNT), Norway, 1995–1997 | Diagnoses in Medical Birth Registry of Norway (MBRN) | Self-reported questionnaires and blood pressure measurement | No information | Age |
| Zhou, 2014[76] | Women delivering at the Guangdong Women and Children's Hospital Affiliated to Guangzhou Medical University, China, January 2008 – June 2012 | Diagnoses in medical records | Blood pressure measurement | 82.8% of preeclampsia and 79.4% of controls complete data | Prenatal, postpartum, and prenatal and postpartum difference in: BMI, blood pressure, fasting blood glucose, fasting insulin, total cholesterol, triglycerides, HDL-C, LDL-C |
| Ghossein-Doha, 2014[40] | Women participating in a previous study in the Netherlands, 1996–1999 | Diagnoses in medical records | Blood pressure measurement | 80% of preeclampsia and 61% of controls complete data | Parity |
| Yeh, 2014[77] | Random selection of women from the National Health Insurance | ICD codes in medical records | ICD codes in medical records | No information | Age, date of delivery |

| | | | | | |
|---|---|---|---|---|---|
| | Research Database (NHIRD) in Taiwan, January 1997 – December 2009 | | | | |
| Breetveld, 2014[41] | Preeclampsia from the 6-week postpartum screening and controls from advertisement, tertiary referral hospital in the Netherlands | Diagnoses in pregnancy charts | No definition of outcome (hypertension) | No information | None |
| Ehrenthal, 2015[65] | Women visiting postpartum service of an academic community hospital, 2011–2012 | Diagnoses in inpatient records | Blood pressure measurement | No information | Age, BMI, race, family history of cardiovascular disease, tobacco use, insurance |
| Behrens, 2016[42] | Women in Denmark with at least one pregnancy, 1978–2012 | ICD codes in National Patient Register and Medical Birth Register | ICD codes in National Patient Register or Causes of Death Register | 97.5% complete data | Maternal birth year, parity, multiple pregnancy, stillbirth |
| Cain, 2016[66] | Singleton first birth to women aged 15–49 years in Florida, January 2004 – December 2007 | ICD codes in Florida's Statewide maternal and infant linked database | ICD codes in hospital discharge data | No information | Age, race/ethnicity, nativity, education, income, history of hyperlipidemia, migraine, and lupus, pre-pregnancy BMI, GDM, tobacco use, drug use, infant sex |
| Nelander, 2016[43] | All twins in the nationwide Swedish Twin Register born in 1958 or earlier, Sweden | Telephone interview | ICD codes in National Patient Register and Cause of Death | No information | Age at interview, current BMI, education, current smoking |

| | | | | | |
|---|---|---|---|---|---|
| | | | Register | | |
| Pérez-Adan, 2016[44] | Women giving birth in the Complexo Hospitalario Universitario de Ourense, Spain, January 2000 – December 2010 | Diagnoses in hospital records | Diagnoses in hospital records | 90.3% complete data | None |
| Grandi, 2017[45] | First birth for women aged 15–45 years in United Kingdom, January 1990 – December 2013 | Read codes in United Kingdom's Clinical Practice Research Datalink (CPRD) | Read codes in United Kingdom's Clinical Practice Research Datalink (CPRD) | No information | Age, smoking, BMI, excessive alcohol use, year of cohort entry, region of residence, multiple gestation at first pregnancy, depression, dyslipidemia, venous thromboembolism, polycystic ovary syndrome, GDM, DM, renal disease, migraines, family history of hypertension and cardiovascular disease, number of distinct drug classes prescribed, use of statin, aspirin, anti-depressant medications, NSAIDs, oral contraceptives, and anti-migraine medications |
| Timpka, 2017[46] | Female registered nurses aged 25–42 years in 1989, United States | Self-reported questionnaires | Self-reports questionnaires | No information | Age, race/ethnicity, parity, BMI at age 18, updated smoking status, alcohol use, NSAIDs use, menopausal status, history of GDM, updated BMI, physical |

| | | | | | |
|---|---|---|---|---|---|
| | | | | | activity, DASH diet, sodium/potassium intake |
| Dunietz, 2017[67] | Women receiving prenatal care from 52 clinics in five Michigan communities, 1998–2004 | Diagnoses in medical records | Blood pressure measurement | 52.1% complete data | Race/ethnicity, pre-pregnancy BMI, age, parity, Medicaid insurance coverage at the time of the Pregnancy Outcomes and Community Health (POUCH) Study pregnancy, time between the POUCH Study pregnancy and follow-up |
| Best, 2017[68] | American Indian women participating in a previous study, January 1995 – December 2012 | Diagnoses in medical records | Diagnoses in medical records | No information | Systolic blood pressure, current age, current BMI |
| Ghossein-Doha, 2017[47] | Preeclampsia from postpartum screening and healthy parous controls from advertisement | Diagnoses in pregnancy charts | Blood pressure measurement | No information | Parity |
| Bokslag, 2018[48] | Women giving birth at two tertiary medical centers in the Netherlands, 1998–2005 | Diagnoses in medical records | Blood pressure measurement | 97.8% of preeclampsia and 91.8% of controls complete data | Age, date of delivery, smoking status, blood pressure, BMI, educational level |
| Li, 2018[78] | Women participating in the Growing Up in Singapore Towards Healthy Outcomes (GUSTO) cohort | Self-reported questionnaires | Blood pressure measurement | 90% complete data | Age, ethnicity, college education, pre-pregnancy BMI, parity |
| Chen, | Women aged 20–50 years admitted | ICD codes in | ICD codes in | No information | Age and year of delivery, parity, |

| | | | | | |
|---|---|---|---|---|---|
| 2018[79] | for delivery in Taiwan, January 2000 – December 2013 | medical records | medical records | | gestational age, gestational number, length of follow-up, comorbidities (DM, hypertension, dyslipidemia, coronary artery disease, chronic kidney disease, COPD, cerebrovascular disease) |
| Bergen, 2018[49] | Women participating in the Generation R Study, Netherlands | Diagnoses in hospital charts | Blood pressure measurement | 97.7% complete data | Age at intake, visit interval, ethnicity, educational level, smoking, subsequent pregnancies between index and follow-up, child's sex, BMI at follow-up |
| Theilen, 2018[69] | Women giving birth in Utah, 1939–2012 | Diagnoses in birth certificate data | Utah Population Database, Utah death certificates, Social Security Death Index | No information | 5-year age groups, year of childbirth, parity, infant sex, gestational age at delivery, parental education, race/ethnicity, marital status |
| Basit, 2018[50] | Women in Denmark with at least one pregnancy, 1978–2015 | ICD codes in National Patient Register | ICD codes in National Patient Register | No information | Age, parity, maternal birth year, region in which the child was delivered |
| Kuo, 2018[80] | Random selection of women from the National Health Insurance Research Database (NHIRD) in Taiwan, January 1996 – December 2010 | ICD codes in medical records | ICD codes in medical records | No information | Age, date of delivery |
| Egeland, | Women participating in the | Diagnoses in | Pharmacologically | No information | Age at delivery, age, BMI, |

| | | | | | |
|---|---|---|---|---|---|
| 2018[51] | Norwegian Mother and Child Cohort Study (MoBa) from all regions in Norway, 1999–2008 | Medical Birth Registry of Norway (MBRN) | treated hypertension in Norwegian Prescription Database | | educational level, physical activity, daily smoking, alcohol consumption frequency, duration of lifetime oral contraceptive use, mid-pregnancy poor diet quality, total energy intake, multiple birth pregnancies at delivery |
| Haas, 2019[70] | First births in 8 United States sites (Case Western University; Columbia University; Indiana University; University of Pittsburgh; Northwestern University; University of California at Irvine; University of Pennsylvania; and University of Utah), 2010–2013 | Diagnoses in medical records | Blood pressure measurement | 79.2% complete data | Age, BMI, type of health insurance at Nulliparous Pregnancy Outcomes Study—Monitoring Mothers-to-be Heart Health Study (nuMoM2b) enrollment, race/ethnicity, smoking during the 3 months before pregnancy |
| Haug, 2019[52] | Women from the Nord-Trøndelag Health Study (HUNT) with at least one birth, Norway, 1967–2012 | Diagnoses in Medical Birth Registry of Norway (MBRN) | Diagnoses in hospital records | 91.7% complete data | Age, maternal birth year, highest educational level, ever daily smoking, parity before age 40 years, family history of coronary heart disease |
| Amiri, 2019[81] | Women aged 20–50 years with at least one pregnancy at the beginning of the Tehran Lipid and Glucose Study (TLGS), Iran, 1998–2014 | Self-reports and diagnoses hospital records | Blood pressure measurement | 95.5% of preeclampsia and 90.0% of controls complete data | None |

| | | | | | |
|---|---|---|---|---|---|
| Smith, 2019[71] | Pre-Eclampsia New Emerging Team (PE-NET) cohort from preeclampsia and normotensive women at the Kingston or Ottawa General Hospitals; Maternal Health Clinic (MHC) cohort from women referred with hypertensive pregnancy, November 2011 – January 2017 | Diagnoses in prenatal records | Blood pressure measurement | No information | Age |
| Honigberg, 2019[53] | Women aged 40–69 years with at least one pregnancy at the baseline of the UK Biobank study visit, 2006–2010 | ICD codes in hospital records or self-reports at enrollment | ICD codes in hospital records and primary care diagnoses | 99.7% complete data | Age, race, BMI, ever-smoking, prevalent hypertension, hyperlipidemia, and DM |
| Ernawati, 2019[82] | Women with severe preeclampsia who carried out pregnancy termination in dr. Soetomo General Hospital of Surabaya, Indonesia, 2013 - January 2014 | Diagnoses in medical records | Blood pressure measurement | 54.8% of early-onset and 39.0% of late-onset complete data | None |
| Osoti, 2019[89] | Women aged 28 years or older delivering at Kenyatta National Hospital, Kemya | Diagnoses in inpatient records | Blood pressure measurement | 91% complete data | Age, level of education, BMI, hormonal contraception, breastfeeding, marital and employment status |
| Leon, 2019[54] | Completed pregnancy registered in United Kingdom's Clinical Practice Research Datalink (CPRD), January 1997 – December 2016 | Read and ICD codes in linked participant records | Read and ICD codes in linked participant records | No information | Ethnicity, age, pre-pregnancy diabetes and hypertension, index of multiple deprivation, cluster term |

| | | | | | |
|---|---|---|---|---|---|
| Arnaout, 2019[72] | First birth to women aged 18 years or older in California, 2005–2009 | ICD codes in Healthcare Cost and Utilization Project (HCUP) database | ICD codes in HCUP database | 99% complete data | Age, race, insurance status, median household income, chronic kidney disease, pre-existing diabetes, obesity, drug abuse, smoking, multiple gestations |
| Huang, 2020[83] | Random selection of women from the National Health Insurance Research Database (NHIRD) in Taiwan, January 2000 – December 2013 | ICD codes in medical records | ICD codes in medical records | No information | Index day, age with propensity scores |
| Scheres, 2020[55] | Women in Denmark with at least one pregnancy, 1999–2012 | Diagnoses in Dutch Perinatal Registry (Perined) | Diagnoses in anticoagulation clinics | 93% complete data | Number of pregnancies, age at start of follow-up, self-reported ancestry |
| Wagata, 2020[84] | Women aged 20 years or older in Miyagi and Iwate Prefectures, Japan, May 2013 – March 2016 | Self-reported questionnaires | Blood pressure measurement | 99.9% complete data | Age, BMI, family history of hypertension, alcohol consumption |
| Garovic, 2020[73] | All live birth or stillborn deliveries in Olmsted County, Minnesota, January 1976 – December 1982 | Rochester Epidemiology Project (REP) medical record-linkage system | Diagnoses in inpatient and outpatient visits to REP-affiliated providers | 98.7% complete data | Date of delivery, age, parity at index pregnancy, education, smoking, obesity |
| Bergman, 2020[56] | All deliveries in Sweden, January 1973 – December 2010 | ICD codes in Swedish Medical | ICD codes in MBR | Complete follow-up | Age at delivery, chronic hypertension before first birth, |

| | | | | | |
|---|---|---|---|---|---|
| | | Birth Register (MBR) | | | level of education, time period of birth |
| Moe, 2020[57] | Women aged 18 years or older delivering a singleton baby at Oslo University Hospital, Norway, September 2014 – April 2018 | Diagnoses in medical records | Blood pressure measurement | Complete follow-up | None |
| Sharma, 2021[58] | Women with pregnancies in the Helsingborg area who delivered at the hospital in the Helsingborg birth cohort (HbgBC) from Sweden linked to registries of the National Board of Health and Welfare in Sweden, February 1964 – January 1967 | ICD codes in national registers | ICD codes in national registers | Complete follow-up | Age at delivery, maternal weight at first antenatal visit, smoking in pregnancy, parity |
| Mooij, 2021[90] | Women who were treated in Ndala Hospital for severe preeclampsia and eclampsia, 2011–2012 | Diagnoses in medical records | Blood pressure measurement | Complete follow-up | None |
| Ntlemo, 2021[91] | Women delivered at two tertiary-level hospitals in Pretoria, February 2019 – April 2020 | Daily morning ward rounds | Blood pressure measurement | 95.8% complete data | None |
| Nuckols, 2021[74] | Women between 18 and 40 years of age, 10–36 months postpartum | Diagnoses in medical records | Blood pressure measurement | Complete follow-up | None |
| Oliver-Williams, 2022[59] | Women who had one or more singleton live births in England recorded in national medical records in all National Health Service | ICD codes in medical records | ICD codes in medical records | No information | Maternal age at delivery, socioeconomic status, ethnicity, hospital-recorded diabetes, diagnosed prior to pregnancy, |

| | | | | | |
|---|---|---|---|---|---|
| | hospitals identified from the Hospital Episode Statistics (HES) database, 1997–2015 | | | | year of delivery |
| Park, 2022[85] | Women who gave birth and recorded in the Korean National Health Insurance Database by the Health Insurance Review & Assessment Service, January 2007 – December 2015 | ICD codes in medical records | ICD codes in medical records | Complete follow-up | Age, primipara, cesarean section, and multiple pregnancy in all data sets; body mass index; systolic and diastolic blood pressures; fasting blood glucose; aspartate aminotransferase levels; alanine aminotransferase levels; total cholesterol levels; and current smoking |
| Hung, 2022[86] | Matching 1:4 selection of women aged 18–45 years without a previous history of stroke registered in the National Health Insurance Research Database (NHIRD) in Taiwan, 2000–2017 | ICD codes in medical records | ICD codes in medical records | No information | Age, delivery type, gestation number, hospital level, delivery season, living area, family income level and all comorbidities |
| Kennedy, 2022[92] | Women gave birth to a live baby at St George Hospital, Sydney, completed at least the 6-month postpartum assessment, and enroll in the subsequent pregnancy component of the P4 study | Diagnoses in inpatient records | Blood pressure measurement | No information | Age and ethnicity, physiological measures (SBP, DBP, fat mass percentage) and interpregnancy factors of BMI change, inter-pregnancy interval and breastfeeding duration |
| van Baar, | Nulliparous women delivered in the | ICD codes in the | ICD codes in the | 96.9% Complete | None |

| | | | | | |
|---|---|---|---|---|---|
| 2022[60] | Netherlands and registered in the Perinatal Registry of the Netherlands (PERINED) database and the National Death Registry of the Netherlands (Central Bureau for Statistics, CBS), 1995–2018 | Perinatal Registry of the Netherlands (PERINED) database | Perinatal Registry of the Netherlands (PERINED) database | data | |

*List of covariates that were adjusted for or matched

Abbreviation: ICD = International Classification of Diseases, GDM = gestational diabetes mellitus, SGA = small-for-gestational age, DM = diabetes mellitus, BMI = body mass index, IUGR = intrauterine growth restriction, LDL-C = low-density lipoprotein cholesterol, HDL-C = high-density lipoprotein cholesterol, hs-CRP = high-sensitivity C-reactive protein, NSAID = non-steroidal anti-inflammatory drug, DASH = diet foods for high blood pressure, COPD = chronic obstructive pulmonary disease; SBP = systolic blood pressure; DBP = diastolic blood pressure

**Supplementary Table 2** Risk of bias assessment of included studies

| Author, year | Representative of the exposed cohort | Selection of the non-exposed cohort | Ascertainment of exposure | Outcome of interest was not present at start of study | Comparability of cohorts | Assessment of outcome | Follow-up long enough | Adequacy of follow-up | Total score |
|---|---|---|---|---|---|---|---|---|---|
| Kestenbaum, 2003[61] | * | * | * | * | * | * | * | | 7 |
| Lykke, 2009[34] | * | * | * | * | * | * | * | * | 8 |
| Edlow, 2009[62] | * | * | * | | * | | | | 4 |
| Garovic, 2010[63] | * | * | * | * | ** | | * | | 7 |
| Mongraw-Chaffin, 2010[64] | * | * | * | * | ** | * | * | | 8 |
| Melchiorre, 2011[35] | * | * | * | | * | | | * | 5 |
| Drost, 2012[36] | * | * | * | | * | * | * | | 6 |
| Zhao, 2012[75] | * | * | * | * | * | * | * | * | 8 |
| Collen, 2013[37] | * | * | | | * | | * | * | 5 |
| Callaway, 2013[87] | * | * | * | * | ** | * | * | | 8 |
| Shalom, 2013[88] | * | * | * | * | | * | * | | 6 |
| Östlund, | * | * | * | * | ** | | * | * | 8 |

| | | | | | | | | | |
|---|---|---|---|---|---|---|---|---|---|
| 2013[38] | | | | | | | | | |
| Kvehaugen, 2014[39] | * | * | * | * | * | | * | | 6 |
| Zhou, 2014[76] | * | * | * | * | * | * | | | 6 |
| Ghossein-Doha, 2014[40] | * | * | * | * | * | * | * | | 7 |
| Yeh, 2014[77] | * | * | * | * | * | * | * | | 7 |
| Breetveld, 2014[41] | * | * | * | * | | | * | | 5 |
| Ehrenthal, 2015[65] | * | * | * | * | ** | * | | | 7 |
| Behrens, 2016[42] | * | * | * | * | * | * | * | * | 8 |
| Cain, 2016[66] | * | * | * | * | ** | * | | | 7 |
| Nelander, 2016[43] | | * | * | | ** | * | * | | 6 |
| Pérez-Adan, 2016[44] | * | * | * | | * | * | * | * | 7 |
| Grandi, 2017[45] | * | * | * | * | ** | * | * | | 8 |
| Timpka, 2017[46] | | * | | * | ** | | | | 4 |
| Dunietz, 2017[67] | * | * | * | | * | * | * | | 6 |

| | | | | | | | | | |
|---|---|---|---|---|---|---|---|---|---|
| Best, 2017[68] | * | * | * | * | ** | * | * | | 8 |
| Ghossein-Doha, 2017[47] | * | * | * | * | | * | * | | 6 |
| Bokslag, 2018[48] | * | * | * | * | ** | * | * | * | 9 |
| Li, 2018[78] | | * | | * | * | * | * | * | 6 |
| Chen, 2018[79] | * | * | * | * | * | * | * | | 7 |
| Bergen, 2018[49] | * | * | * | * | ** | * | * | * | 9 |
| Theilen, 2018[69] | * | * | * | * | * | * | * | | 7 |
| Basit, 2018[50] | * | * | * | * | * | * | * | | 7 |
| Kuo, 2018[80] | * | * | * | * | * | * | * | | 7 |
| Egeland, 2018[51] | * | * | * | * | ** | * | * | | 8 |
| Haas, 2019[70] | * | * | * | * | ** | * | * | | 8 |
| Haug, 2019[52] | * | * | * | * | * | * | * | * | 8 |
| Amiri, 2019[81] | * | * | | * | | * | * | * | 6 |
| Smith, 2019[71] | * | | * | | * | * | | | 4 |
| Honigberg, 2019[53] | * | * | * | * | ** | * | * | * | 9 |
| Ernawati, 2019[82] | * | * | * | * | * | * | * | | 7 |
| Osoti, 2019[89] | * | * | * | * | * | * | | * | 7 |

| | | | | | | | | | |
|---|---|---|---|---|---|---|---|---|---|
| Leon, 2019[54] | * | * | * | * | * | * | * | | 7 |
| Arnaout, 2019[72] | * | * | * | * | ** | * | | * | 8 |
| Huang, 2020[83] | * | * | * | * | * | * | * | | 7 |
| Scheres, 2020[55] | * | * | * | * | * | * | * | * | 8 |
| Wagata, 2020[84] | * | * | | | * | * | * | * | 6 |
| Garovic, 2020[73] | * | * | * | * | ** | * | * | * | 9 |
| Bergman, 2020[56] | * | * | * | * | * | * | * | * | 8 |
| Moe, 2020[57] | * | * | * | * | * | * | | * | 7 |
| Sharma, 2021[58] | * | * | * | * | ** | * | * | * | 9 |
| Mooij, 2021[90] | | * | * | * | | * | * | * | 6 |
| Ntlemo, 2021[91] | * | * | * | * | | * | | * | 6 |
| Nuckols, 2021[74] | * | * | * | * | | * | | * | 6 |
| Oliver-Williams, 2022[59] | * | * | * | * | ** | * | * | | 8 |
| Park, 2022[85] | * | * | * | * | ** | * | * | * | 9 |

| | | | | | | | | | |
|---|---|---|---|---|---|---|---|---|---|
| Hung, 2022[86] | * | * | * | * | * | * | * | | 7 |
| Kennedy, 2022[92] | * | * | * | * | ** | * | | | 7 |
| van Baar, 2022[60] | * | * | * | * | | * | * | * | 7 |

**Supplementary Table 3** Summary of findings table for each outcome

| Outcomes | RR (95% CI) | Certainty of evidence |
|---|---|---|
| **Prior HDP vs normotensive pregnancies** | | |
| Hypertension at any time (n= 37) | | ⊕⊕⊖⊖ Low[1,2,3,4] |
| Hypertension <= 5 years (n = 7) | 5.34 (2.74, 10.39) | ⊕⊖⊖⊖ Very low[1,3,4] |
| Hypertension 6-10 years (n = 6) | 4.22 (2.19, 8.10) | ⊕⊖⊖⊖ Very low[1,3,4] |
| Hypertension 11-15 years (n = 7) | 3.27 (2.02, 5.30) | ⊕⊕⊖⊖ Low[1,2,3,4] |
| Hypertension >=15 years (n = 2) | 1.79 (1.22, 2.61) | ⊕⊕⊖⊖ Low[1,4] |
| Hypertension unspecified (n =15) | 3.10 (1.81. 5.29) | ⊕⊕⊖⊖ Low[1,3] |
| Ischemic heart disease (n = 10) | 2.06 (1.38, 3.08) | ⊕⊕⊖⊖ Low[1,3] |
| Heart failure (n = 7) | 2.53 (1.28, 5.00) | ⊕⊖⊖⊖ Very low[1,3,4] |
| Venous thromboembolism (n = 4) | 1.34 (0.80, 2.24) | ⊕⊖⊖⊖ Very low[1,3,4,5] |
| Peripheral vascular disease (n = 3) | 1.23 (0.26, 5.76) | ⊕⊖⊖⊖ Very low[1,4,5] |
| Stroke (n = 12) | 1.59 (1.08, 2.33) | ⊕⊕⊖⊖ Low[1,3] |
| Dementia (n = 3) | 1.37 (0.70, 2.71) | ⊕⊖⊖⊖ Very low[1,4,5] |
| Composite cardiovascular and cerebrovascular diseases (n = 8) | 1.91 (1.18, 3.09) | ⊕⊖⊖⊖ Very low[1,3,4,5] |
| Mortality (n = 2) | 2.15 (0.70, 6.58) | ⊕⊖⊖⊖ Very low[1,3,4,5] |
| **Preeclampsia with severe features vs normotensive pregnancies** | | |
| Hypertension at any time (n = 3) | 6.67 (1.51, 29.40) | ⊕⊖⊖⊖ Very low[1,3,4,5] |
| Ischemic heart disease (n = 2) | 2.11 (0.04, 113.97) | ⊕⊖⊖⊖ Very low[1,3,4,6] |
| Venous thromboembolism (n = 2) | 2.00 (0.41, 9.78) | ⊕⊖⊖⊖ Very low[1,4,6] |
| **Preeclampsia without severe features vs normotensive pregnancies** | | |
| Hypertension at any time (n = 2) | 7.21 (0, 17565) | ⊕⊖⊖⊖ Very low[1,3,4,6] |
| Ischemic heart disease (n = 2) | 1.92 (1.09, 3.38) | ⊕⊖⊖⊖ Very low[1,4,5] |
| Venous thromboembolism (n = 2) | 1.64 (1.55, 1.73) | ⊕⊕⊖⊖ Low[1,4] |
| **Early onset preeclampsia vs normotensive pregnancies** | | |
| Hypertension at any time (n = 2) | 2.53 (1.93, 3.32) | ⊕⊕⊖⊖ Low[1,4] |
| The corresponding risk (and its 95% confidence interval) is based on the assumed risk in the | | |

| |
|---|
| comparison group and the relative effect of the intervention (and its 95% CI). CI: Confidence interval; RR: Relative Risk |
| GRADE Working Group grades of evidence<br>• High quality: Further research is very unlikely to change our confidence in the estimate of effect.<br>• Moderate quality: Further research is likely to have an important impact on our confidence in the estimate of effect and may change the estimate.<br>• Low quality: Further research is very likely to have an important impact on our confidence in the estimate of effect and is likely to change the estimate.<br>• Very low quality: We are very uncertain about the estimate. |
| Footnotes<br>[1]We downgraded (1) level for serious limitation in study design.<br>[2]We upgraded (1) level for magnitude of effect.<br>[3]We downgraded (1) level for serious inconsistency.<br>[4]We downgraded (1) level for serious publication bias due to p value <0.05.<br>[5]We downgraded (1) level for serious imprecision due to wide confidence interval.<br>[6]We downgraded (2) level for very serious imprecision due to wide confidence interval. |
